# Beneficial effects of hypotaurine supplementation in preparation and freezing media on human sperm cryo-capacitation and DNA quality

**DOI:** 10.1186/s12610-021-00144-6

**Published:** 2021-11-04

**Authors:** Hanae Pons-Rejraji, Solène Vorilhon, Asmaa Difrane, Sandra Dollet, Céline Bourgne, Marc Berger, Laure Chaput, Bruno Pereira, Cyril Bouche, Joël R. Drevet, Florence Brugnon

**Affiliations:** 1 CHU Clermont Ferrand, CHU Estaing, Assistance Médicale à la Procréation – CECOS, F-63003 Clermont-Ferrand, France; 2 Université Clermont Auvergne, INSERM 1240, IMoST, F-63000 Clermont-Ferrand, France; 3 CHU Clermont Ferrand, CHU Estaing, Laboratoire d’Hématologie Biologique, F-63003 Clermont-Ferrand, France; 4 CHU Clermont-Ferrand, DRCI, Biostatistics Unit ‘Délégation Recherche Clinique et Innovation’, Clermont-Ferrand, France; 5 Université Clermont Auvergne, CNRS UMR6293, INSERM U1103, GReD, F-63000 Clermont-Ferrand, France

**Keywords:** Cryopreservation, Human spermatozoa, Density gradient centrifugation, DNA fragmentation and oxidation, Chromatin packaging, Acrosome, Capacitation and cryocapacitation, cryoconservation, spermatozoïdes humains, test de migration survie, fragmentation et oxydation de l’ADN, condensation de la chromatine, réaction acrosomique, capacitation et cryocapacitation

## Abstract

**Background:**

Although widely used, slow freezing considerably modifies the functions of human spermatozoa. Cryopreservation induces nuclear sperm alterations and cryo-capacitation, reducing the chances of pregnancy. Hypotaurine is naturally present in the male and female genital tracts and has capacitating, osmolytic and anti-oxidant properties. The analysis were performed on surplus semen of men with normal (*n* = 19) or abnormal (*n* = 14) sperm parameters. Spermatozoa were selected by density gradient centrifugation before slow freezing. For each sample, these steps were performed in parallel with (“H+” arm) or without (“H-” arm) hypotaurine supplementation. After thawing, we measured total and progressive mobility, vitality, acrosome integrity, markers of capacitation signaling pathway and nuclear quality. For the latter, we focused on sperm chromatin packaging, DNA fragmentation and the presence of vacuoles in the sperm nucleus.

**Results:**

Post-thaw spermatozoa selected and frozen in the presence of hypotaurine had a higher vitality (+ 16.7%, *p* < 0.001), progressive and total motility (+ 39.9% and +  21.6% respectively, *p* < 0.005) than spermatozoa from the control “H-” arm. Hypotaurine also reduced the non-specific phosphorylation of the capacitation protein markers P110 and P80 (*p* < 0.01), indicating a decrease in cryo-capacitation. Hypotaurine supplementation reduced chromatin decondensation, measured by chromomycin A3 (− 16.1%, *p* < 0.05), DNA fragmentation (− 18.7%, *p* < 0.05) and nuclear vacuolization (− 20.8%, *p* < 0.05).

**Conclusion:**

Our study is the first to demonstrate beneficial effects of hypotaurine supplementation in preparation and freezing procedures on human spermatozoa sperm fertilization capacity and nucleus quality. Hypotaurine supplementation limited cryo-capacitation, increased the proportion of live and progressively motile spermatozoa and reduces the percentage of spermatozoa showing chromatin decondensation, DNA fragmentation and nuclear vacuolation.

**Trial registration:**

Clinical Trial, NCT04011813. Registered 19 May 2019 - Retrospectively registered.

## Résumé

**Contexte:**

Bien que largement utilisée, la congélation lente modifie considérablement les fonctions des spermatozoïdes humains. La cryoconservation induit des altérations nucléaires du sperme et une cryocapacitation, réduisant les chances de grossesse. L’hypotaurine est. naturellement présente dans les voies génitales masculines et féminines et possède des propriétés capacitantes, osmotiques et anti-oxydantes. Les mesures ont été réalisées sur le reliquat de sperme d’hommes avec des paramètres spermatiques normaux (*n* = 19) ou anormaux (*n* = 14). Les spermatozoïdes ont été sélectionnés par centrifugation sur gradient de densité (test de migration survie) avant congélation lente. Pour chaque prélèvement, ces étapes ont été réalisées en parallèle avec des milieux supplémentés en hypotaurine (bras « H+ ») ou sans hypotaurine (bras « H- »). Après décongélation, nous avons mesuré la mobilité totale et progressive, la vitalité, l’intégrité de l’acrosome, des marqueurs de la voie de signalisation de la capacitation et la qualité nucléaire. Pour cette dernière, nous nous sommes concentrés sur la condensation de la chromatine, la fragmentation de l’ADN et la présence de vacuoles dans le noyau du sperme.

**Résultats:**

Post-décongélation, les spermatozoïdes sélectionnés et congelés en présence d’hypotaurine avaient une vitalité plus élevée (+ 16,7%, *p* < 0,001), une motilité progressive et totale (+ 39,9% et + 21,6% respectivement, *p* < 0,005) que les spermatozoïdes du bras « H- » sans suplémentation. L’hypotaurine a également réduit la phosphorylation non spécifique des marqueurs protéiques de capacitation P110 et P80 (*p* < 0,01), indiquant une diminution de la cryocapacitation. La supplémentation en hypotaurine a réduit la décondensation de la chromatine, mesurée par la chromomycine A3 (− 16,1%, *p* < 0,05), la fragmentation de l’ADN (− 18,7%, *p* < 0,05) et la vacuolisation nucléaire (− 20,8%, p < 0,05).

**Conclusion:**

Notre étude est. la première à démontrer les effets bénéfiques de la supplémentation en hypotaurine dans les milieux de préparation et de congélation sur la capacité de fécondation des spermatozoïdes humains et leur qualité nucléaire. La supplémentation en hypotaurine a limité la cryocapacitation, augmenté la proportion de spermatozoïdes vivants et progressivement mobiles et réduit le pourcentage de spermatozoïdes présentant une décondensation de la chromatine, une fragmentation de l’ADN et une vacuolisation nucléaire.

**Enregistrement de l’essai:**

essai clinique, NCT04011813. Enregistré le 19 mai 2019 - Enregistré rétrospectivement.

## Background

Despite advances in cryobiology, slow freezing remains the standard method of cryopreservation of human spermatozoa [1–3]. Nevertheless, this method considerably affects sperm integrity, decreasing vitality and motility, and inducing both structural and functional changes, resulting in decreased sperm fertilizing capacity [4–7]. Freeze-thaw cycles also increase the production of reactive oxygen species (ROS) and reduce the amount and activity of antioxidant (AO) systems, leading to oxidative stress (OS, for reviews see [8–10]). The effects of the latter are well known: disruption of plasma, acrosomal and mitochondrial membranes; impaired motility and fertilisation capacity; and, finally, sperm death, especially by apoptosis [1, 10–12]. OS is also the main source of DNA damage, resulting in base oxidation, DNA fragmentation and chromatin packaging disorders [13, 14]. Previous studies have reported, after thawing a higher percentage of spermatozoa without acrosomes or with an altered acrosomal membrane, and an increased proportion of spermatozoa with cryo-capacitation characteristics. These sperm alterations are associated with premature capacitation or acrosome reaction or both, with low fertilization capacity and limited survival time [6, 15–17].

To reduce the deleterious effects of the freeze-thaw cycle, two main strategies have been reported. The first is the selection of high-quality spermatozoa, notably by density gradient centrifugation (DGC) or swim up [5, 18]. This technique removes the most immature germ cells, immune or epithelial cells, and dead or abnormal spermatozoa, thereby helping to reduce sources of ROS. However, the removal of seminal fluid deprives sperm cells of their natural AO protection [19], which has a detrimental effect, especially on abnormal semen, as has been reported recently [20]. The second strategy is more common and consists of providing AO to protect spermatozoa [21–27]. Few studies have shown the beneficial effect of combining the two strategies [28, 29]. In one such study [28], semen samples from patients with oligo-astheno-teratozoospermia (OAT) were selected by DGC and supplemented with hypotaurine, which was also added to the washing and cryopreservation media. We showed beneficial effects, including a decrease in the proportion of spermatozoa with phosphatidylserine externalization, an increase in spermatozoa mitochondrial potential and a decrease in DNA fragmentation.

In order to substantiate the beneficial effects of such a strategy, the objective of this study was to investigate further the effects of hypotaurine supplementation on a wide range of sperm parameters, with particular emphasis on sperm fertilizing ability and DNA quality. To this end, we simultaneously evaluated: (i) conventional sperm parameters; (ii) fertilization capacity by measuring acrosome integrity, the level of cryocapacitated spermatozoa after thawing and the ability of sperm cells to capacitate in vitro; (iii) sperm nuclear alterations such as chromatin decondensation, DNA fragmentation and the presence of vacuoles in the sperm heads (i.e. nuclear vacuolation)*.*

## Materials and methods

### Experimental design, ethical statement and patient characteristics

**Fig. 1:**
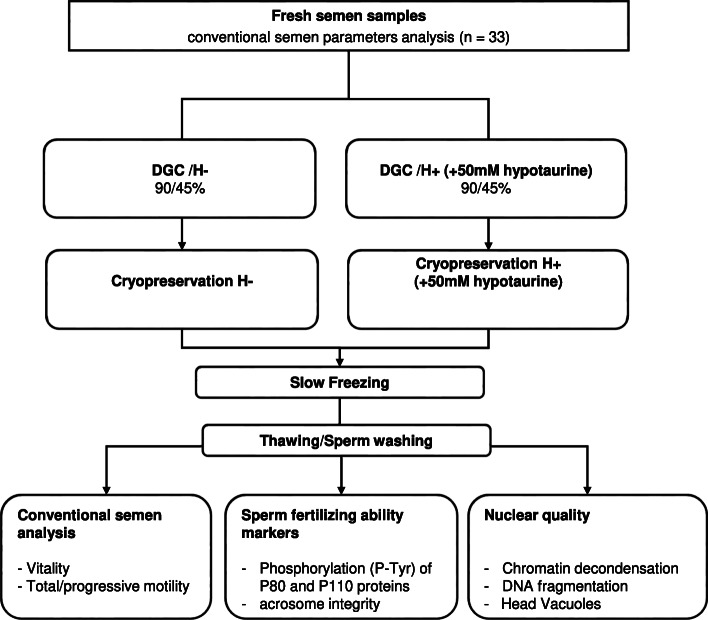
Experimental study design. Samples were obtained from 33 men undergoing routine semen analysis. Excess semen from each sample was split in two and processed in parallel in two ways: a control arm “H -”: sperm cells were processed by density gradient centrifugation (DGC) without hypotaurine supplementation, and an “H +” arm: spermatozoa were processed by 50 mM hypotaurine supplementation during DGC. Spermatozoa were washed and frozen in high-security straws in the presence of hypotaurine supplemented (H+) or non-hypotaurine supplemented (H-) media. After thawing, the different analyzes were carried out in parallel for both conditions: - (i) Analysis of standard semen parameters (*n* = 33): Sperm vitality,progressive and total motilities. - (ii) Measurements of fertilization capacity: Evaluation of (cryo-) capacitation terminal markers by measuring tyrosine phosphorylation of P110 and P80 (*n* = 15) and analysisof acrosome integrity after FITC- PSA labeling (*n* = 25), and - (iii) Measurements of nuclear quality markers: Chromatin condensation measured by chromomycin A3 (*n* = 28), DNA fragmentation by TUNEL assay (*n* = 18), and nuclear vacuolization observed by examination of motile sperm organelle morphology (MSOME, *n* = 19)

The experimental design is shown in Fig. 1. Samples were obtained from 33 men undergoing routine semen analysis at the Center for Reproductive Medicine. Semen samples were collected and conventional analyses were performed in accordance with World Health Organization (WHO) 2010 guidelines [30]. Each sperm sample was split in two and processed in parallel in two ways: an “H^−^” control arm: DGC before freezing without hypotaurine supplementation and an “H^+^” arm: DGC before freezing with a 50 mM hypotaurine supplementation in DGC, washing and freezing media. Spermatozoa were frozen in high-security straws (CBS™, Crybiosystem, L’Aigle, France) in the presence of Cryosperm® freezing medium (Origio, Malov, Denmark) and stored at − 196 °C in liquid nitrogen until use. After thawing, the analyses were performed in parallel for the two conditions. These included: (i) standard semen parameter evaluation, (ii) sperm fertilizing capacity, and (iii) sperm nuclear quality. Thirty-three patients with a mean age of 33.5 ± 0.8 years (Table 1) were included in the study. The mean body mass index (BMI) was 26.2 ± 0.8 (Table 1). Semen parameters are presented in Tables 1, 19 samples were normozoospermic and 14 had abnormal semen parameters including teratozoospermia, asthenozoospermia and high level of leukocytes.

**Table 1:** Clinical characteristic and standard semen parameters

	All patients	Normo-zoospermic patients	Patients with abnormal sperm parameters
Number of subjects	33	19	14
Age (years)	33.5 ± 0.8	34.0 ± 1.1	32.9 ± 1.2
BMI	26.2 ± 0.8	26.3 ± 1.0	26.1 ± 1.3
**Standard semen parameters**			
Semen volume (ml)	4.7 ± 0.4	4.6 ± 0.3	4.8 ± 0.8
pH	7.9 ± 0.05	7.9 ± 0.03	7.8 ± 0.1
Spz concentration (10^6^ cells/ml)	112.8 ± 13.3	119.7 ± 14.5	103.5 ± 24.7
Round cells concentration (10^6^ cells/ml)	3.0 ± 0.6	2.5 ± 0.6	3.8 ± 1.0
PMN concentration (10^6^ cells/ml)	0.1 ± 0.1	0.1 ± 0.1	0.2 ± 0.2
Vitality (%)	73.6 ± 1.9	75.0 ± 1.9	71.4 ± 3.8
Total motility (%)	54.3 ± 1.6	55.5 ± 1.9	52.7 ± 3.0
Progressive motility (%)	46.1 ± 1.7	47.7 ± 2.1	43.8 ± 2.7
Typical morphology (%)	7.6 ± 1.5	12.3 ± 1.8	1.3 ± 0.3

Values are represented as mean ± SEM. Spermatozoa morphology was measured using a stricter criteria classification and conventional semen parameters were established according to the WHO 2010 guidelines. *BMI* body mass index. *Spz* spermatozoa. *PMN* PolyMorphonuclear Neutrophil

### Chemicals

All chemicals were purchased from Sigma-Aldrich (St-Quentin-Fallavier, France) unless otherwise indicated.

### Semen collection and preparation

Semen samples were collected by masturbation in sterile containers after a period of 2–7 days of sexual abstinence. Conventional semen analysis were performed in accordance with the WHO 2010 guidelines [30]. All sperm counts are the result of two independent readings. Leukocytes were detected using the LeucoScreen kit (FertiPro N.V., Beernem, Belgium). The proportion of viable spermatozoa (vitality) was estimated by assessing sperm membrane integrity by eosin-nigrosin staining using the Vital Screen Kit (FertiPro N.V.). Morphology was estimated according to Krüger’s stricter criteria [31]. Surplus semen was processed by a two-stage (90/45%), 750 g, 20 min, discontinuous density gradient centrifugation (DGC) at room temperature (RT) using a fresh gradient medium consisting of Puresperm® medium (Nidacon International, Molndal, Sweden) diluted with balanced Sperm Preparation® medium (Origio, Malov, Denmark). The high density fraction was then washed by centrifugation in Sperm Preparation® medium at 750 g, for 8 min at RT and the pellet was resuspended in the washing medium. Samples were mixed in a 1:1 ratio (v/v) with a cryoprotective medium (Cryosperm, Origio, Malov, Denmark) according to the supplier’s recommendations. Afterwards sperm samples were frozen in high security straws (Cryobiosystem®) in a NanoDigitcool® programmable freezer (Cryobiosystem®) and stored in a liquid nitrogen tank (CF250M, Cryodiffusion®, Lery, France) as previously described [28]. For the H+ arm, all media were supplemented with a final concentration of 50 mM of hypotaurine as previously described [28].

For thawing, the straws were placed at 37 °C for 3 min. After a multi-step addition of Sperm Preparation® medium, the cryoprotectant was removed by centrifugation (750 g, 5 min, RT). Finally, a second wash was performed with Sperm Preparation® medium (750 g, 5 min, RT) and the samples were dispatched for evaluation.

### Fertilizing ability measurements

#### Western blot of the capacitation terminal markers

Sperm capacitation and cryo-capacitation were estimated by monitoring tyrosine phosphorylation (P-Tyr) of the P80 and P110 proteins by the western-blot procedure (*n* = 15), by using an anti-phosphotyrosine monoclonal antibody (clone 4G10; Upstate Biotechnology Inc., Lake Placid, NY). For all the samples, the P-Tyr of P80 and P110 were evaluated in spermatozoa directly after thawing, indicating basal phosphorylation of both markers due to cryo-capacitation, and after 3 h incubation under capacitating conditions (Sperm Preparation medium, 37 °C and 5% CO_2_) corresponding to effective capacitation. Incubations under capacitating conditions and immunoassays were performed as described in previous studies [32]. The same membranes were probed again using the same protocol with a mouse anti α-tubulin monoclonal antibody. P-Tyr profiles and tubulin signals were quantified and the P-Tyr signal was normalized to that of tubulin. For each condition, the ratio obtained after different incubation times was related to the ratio obtained before incubation.

#### Acrosomal integrity measurements

For the evaluation of acrosomal integrity, spermatozoa from 25 samples were fixed and permeabilized in methanol 98% after washing with Phosphate Buffer Saline (PBS) 1X. Sperm cells were incubated with a freshly prepared solution of *P. sativum* agglutinin conjugated to fluorescein isothiocyanate (FITC-PSA, 60 μg/ml final) in PBS 1X for 30 min at 4 °C in a humid chamber as indicated in prior studies [33]. After washing, the spermatozoa were observed in an epifluorescence microscope (× 400). Sperm cells with their acrosomal membranes showed marked fluorescence on the acrosomal region, while those that had lost their acrosomal membrane (AR for acrosome reacted) were either devoid of fluorescence or showed a fluorescence mark along the equatorial segment. A total of 200 spermatozoa *per* slide were counted blind twice for each condition.

### Measurement of nuclear quality markers

#### Chromatin packaging measurements

Chromatin decondensation measurements were performed by using chromomycin A3 labelling (*n* = 28). Spermatozoa were fixed in methanol/glacial acetic acid (3:1) at 4 °C for 5 min, before being labelled with CMA3 solution (0.25 mg/ml of McIlvaine buffer, pH 7.0 and 10 mM of MgCl_2_) in a humid chamber. Bright yellow sperm heads are a sign of abnormally condensed chromatin. A total of 200 spermatozoa per slide were counted blind twice for each condition.

#### DNA fragmentation measurements

Sperm DNA fragmentation (*n* = 19) was measured by using the TUNEL detection Kit (Cell Death Kit detection POD®, Roche, France) as previously described [28]. After washing by centrifugation (500 g, 5 min, TA, PBS 1X), 1.10^6^ spermatozoa were fixed in 4% (w/v) paraformaldehyde. After being washed in PBS 1X-BSA 1%, sperm samples were resuspended in a permeabilization solution containing 0.1% (v/v) Triton-X and 0.1% (w/v) sodium citrate for 3 min in ice. After washing (500 g, 5 min, RT), the labeling reaction was carried out by incubating for 1 h at 37 °C with the enzyme and the labeling solution. The cell suspension was then washed in 1% (v/v) PBS-BSA and counter-stained with propidium Iodide (PI, 2 mg/ml) to check the effectiveness of the permeabilization before flow cytometric (FCM) measurements.

#### Flow Cytometry measurements

Flow Cytometry measurements were performed on a minimum of 20,000 spermatozoa using a BD FACS Aria SORP cell sorter (BD Biosciences, Franklin Lakes, NJ) with an 85 μm nozzle. The FSC (forward-scattered light) and SSC (side-scattered light) were detected on a linear scale and used to gate cells. The fluorochromes were excited at a wavelength of 488 nm by an argon laser. Green (FITC) and red (PI) fluorescence were detected at FL1 by a 525 nm bandwidth filter and at FL3 by a 620 nm bandwidth filter. The fluorescence signals detected at FL1 and FL3 were recorded after logarithmic amplification and the percentage of marked cells was calculated by the flow cytometer software. Sperm gating was confirmed by microscopy after cell sorting.

#### Nuclear vacuolization analysis

Nuclear vacuolization was estimated for 19 samples by motile sperm organelle morphology examination (MSOME) in an inverted microscope (Leica AM 6000 micromanipulator, Nanterre, France) equipped with a Hoffman and Nomarski interferential contrast with a dry objective of × 6300. Sperm suspensions were observed in a microdrop of 5 μl PVP (Polyvinylpyrrolidone, Clinical Grade®, Origio, Malov, Denmark) in a sterile glass dish covered with mineral oil (Ovoil®, Vitrolife, Västra Frölunda, Sweden). The spermatozoa were classified into 2 groups: (i) those with a large head vacuole or 2 or more small vacuoles; (ii) a normal group of those without a vacuole or with one small nuclear vacuole. A total of 200 spermatozoa were counted twice blind for each condition.

### Statistics

All statistical analyses were performed using STATA® software (version 13, StataCorp, College Station, Lakeway Drive, USA). Data are presented as mean ± standard error of the mean (SEM). For each paired comparison, the statistical difference was evaluated using paired parametric (Student’s t-test) or non-parametric (Wilcoxon test) tests. The assumption of normality was studied using Shapiro-Wilk’s test and the homoscedasticity was analyzed by the use of Pitman’s test. The results were expressed using effect-size (ES) values with 95% confidence interval (C.I.) [34]. Subgroups analyses of abnormal and normal samples were carried out for the parameters for which the sample number in the both groups was greater than or equal to 10, that is to say mobility, vitality, DNA compaction and acrosome integrity. The relationships between continuous parameters were evaluated by Pearson or Spearman correlation coefficients (noted rho coefficient) according to the statistical distribution and applying a Sidak’s type I error correction. Results were represented using a color-coded heatmap in order to illustrate the different correlations.

## Results

### Sperm vitality and motility improvement

Sperm vitality, total and progressive motility were significantly reduced after thawing compared with fresh sperm values (Table 1) for both H- (*p* < 0.0001 for all three parameters) and H+ (p< 0.0001 for vitality, p<0.01 and *p* < 0.05 for total and progressive motility respectively) arms (Table 2). These three parameters were significantly higher for the H+ condition than H- one (+ 16.6% *p* < 0.001 for vitality, + 39.8% and +  21.8% *p* < 0.005 for progressive and total motility, Table 2, all patients). Hypotaurine supplementation was particularly beneficial on abnormal sperm samples (+ 20.4%, ES = 0.40 [0.10; 0.74], + 32.3%, ES = 0.48 [0.05; 1.05]; 40.3%, ES = 0.54 [0.01; 1.13] for vitality and progressive and total motility respectively, Table 2).

**Table 2:** Effects of hypotaurine supplementation on standard semen parameters

	H-	H+	ES [C.I.]
Vitality (%, *n* = 33)	39.0 ± 2.9	**45.5** ± 3.2 (+ 16.7%) ***	**0.35 [0.16; 0.57]**
Normozoospermic patients (*n* = 19)	39.2 ± 3.8	**44.7** ± 4.3 (+ 14.0%) *	**0.29 [0.03; 0.59]**
Patients with abnormal sperm (*n* = 14)	38.7 ± 4.8	**46.6** ± 5.0 (+ 20.4%) *	**0.40 [0.10; 0.74]**
Total motilty (%, *n* = 33)	36.5 ± 3.5	**44.4** ± 3.6 (+ 21.6%) **	**0.38 [0.07; 0.69]**
Normozoospermic patients (*n* = 19)	37.1 ± 4.5	42.3 ± 4.3 (+ 14.0%)	0.26 [0.10; 0.63]
Patients with abnormal sperm (*n* = 14)	35.6 ± 5.7	**47.1** ± 6.4 (+ 32.3%) *	**0.54 [0.01; 1.13]**
Progressive motility (%, *n* = 33)	17.3 ± 2.1	**24.2** ± 2.3 (+ 39.9%) **	**0.53 [0.15; 0.94]**
Normozoospermic patients (*n* = 19)	18.7 ± 2.8	**26.2** ± 3.5 (+ 40.1%) **	**0.51 [0.01; 1.04]**
Patients with abnormal sperm (*n* = 14)	15.4 ± 3.1	**21.6** ± 2.8 (+ 40.3%) *	**0.48 [0.05; 1.05]**

Values are represented as mean ± SEM for percentage (%). “H -”: sperm cells were processed by density gradient centrifugation (DGC), washed and frozen without hypotaurine supplementation. “H +”: sperm cells were processed by DGC, washed and frozen in the presence of 50 mM hypotaurine. After thawing the percentage of viable (vitality), motile (total and progressive motility) spermatozoa was measured. H+ and H- conditions were compared using paired Student t-test or non-parametric test when assumptions of t-test were not met. The results were also expressed using effect-size (ES) and 95% confidence interval (C.I.). *, **, *** indicate a significant difference with *p* < 0.05, *p* < 0.01 and *p* < 0.001 in comparison with condition H-

### Sperm cryo-capacitation limitation

**Fig. 2:**
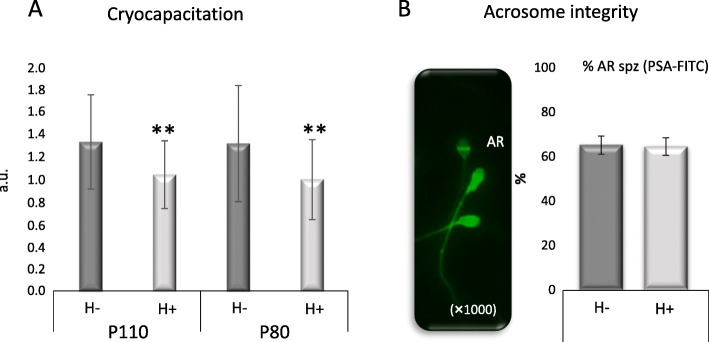
Effects of hypotaurine supplementation on sperm fertilization capacity after thawing. Numbers are averaged ± SEM for (**A**) P110 and P80 (arbitrary units: a.u.) and percent ± SEM for (**B**) acrosome integrity (%). “H -”: sperm cells were processed by density gradient centrifugation (DGC), washed and frozen without hypotaurine supplementation. “H +”: sperm cells were processed by DGC, washed and frozen in the presence of 50 mM of hypotaurine. After thawing, measurements were performed on (**A**) the levels of cryo-capacitation (n = 15), and (**B**) the percentage of altered or reacted (AR) spermatozoa (n = 25). Cryo-capacitation levels were evaluated by measuring the tyrosine phosphorylation of proteins P110 and P80 after thawing under capacitation conditions (37 °C and 5% CO_2_). Conditions H+ and H- were compared using parametric or non-parametric tests based on statistical distribution and number of subjects (n).** indicates significant difference with p < 0.01 in comparison to the H- condition

After thawing, we observed lower levels of phosphorylation of P110 and P80 proteins for the H+ condition, indicating a lower level of cryo-capacitation than the H- one (*p* < 0.01 for both P110 and P80, Fig. 2A). After 3 h of incubation under capacitation conditions, the phosphorylation levels of P110 and P80 tended to be higher for the H+ condition compared to the H- one, although this was not statistically significant for either P110 (3.5 ± 1.0 for H- vs. 4.4 ± 1.0 a.u. for H+, *p* = 0.23 Fig. 2A) or for P80 (3.6 ± 0.9 for H- vs. 4.5 ± 1.0 a.u. for H+, *p* = 0.08). We did not measure a significant difference between the two conditions with respect to the percentage of spermatozoa with altered or absent acrosomes (65.1 ± 3.2 for H- vs. 64.4 ± 3.1% for H+, AR %, Fig. 2B). No significant effect was measured either for normal (60.0 ± 4.6 for H- vs. 60.6 ± 4.5% for H+ for) or for abnormal (71.7 ± 3.5 for H- vs. 69.3 ± 3.7% for H+) sperm samples.

### Sperm nuclear protection

**Fig. 3:**
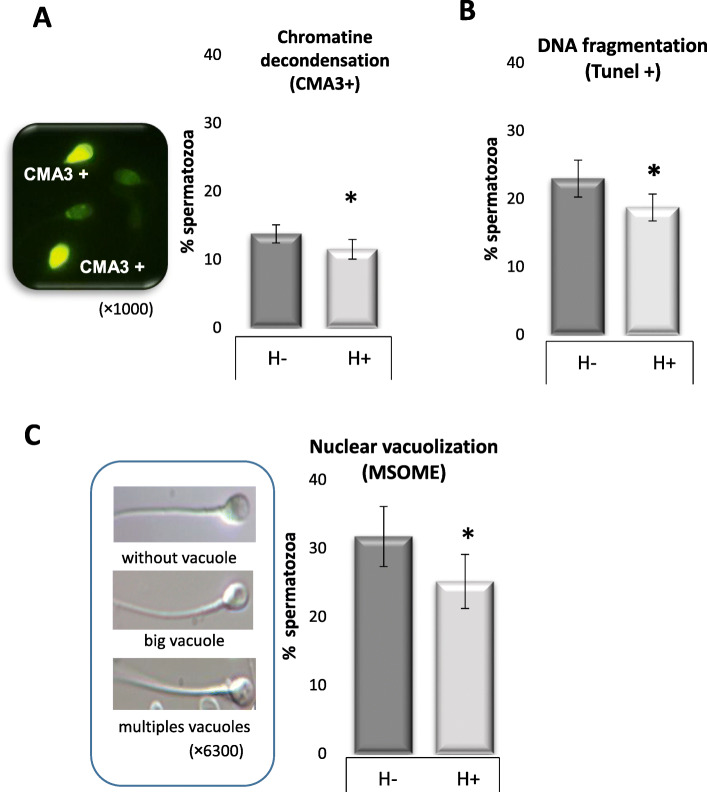
Effects of hypotaurine supplementation on sperm nuclear quality after thawing. Values are mean ± SEM for the percentage (%) of spermatozoa with (**A**) decondensed chromatin, (**B**) fragmented DNA or (C) head vacuoles.”H -”: sperm cells were processed by density gradient centrifugation (DGC), washed and frozen without hypotaurine supplementation. “H +”: sperm cells were processed by DGC, washed and frozen in the presence of 50 mM hypotaurine. The following were measured after thawing: (**A**) Chromatin packaging: percentage of spermatozoa with decondensed chromatin evaluated by chromomycine A3 (% CMA3 + spermatozoa, n = 28); (**B**) DNA fragmentation estimated by the TUNEL assay (% TUNEL + spermatozoa, n = 19). (**C**) nuclear vacuolization estimated by MSOME (% spermatozoa with head vacuoles, n = 19). Conditions H+ and H- were compared using parametric or non-parametric tests according to statistical distribution and the number of subjects (n).* indicates a significant difference with *p* < 0.05 in comparison to the H- condition

**Fig. 4:**
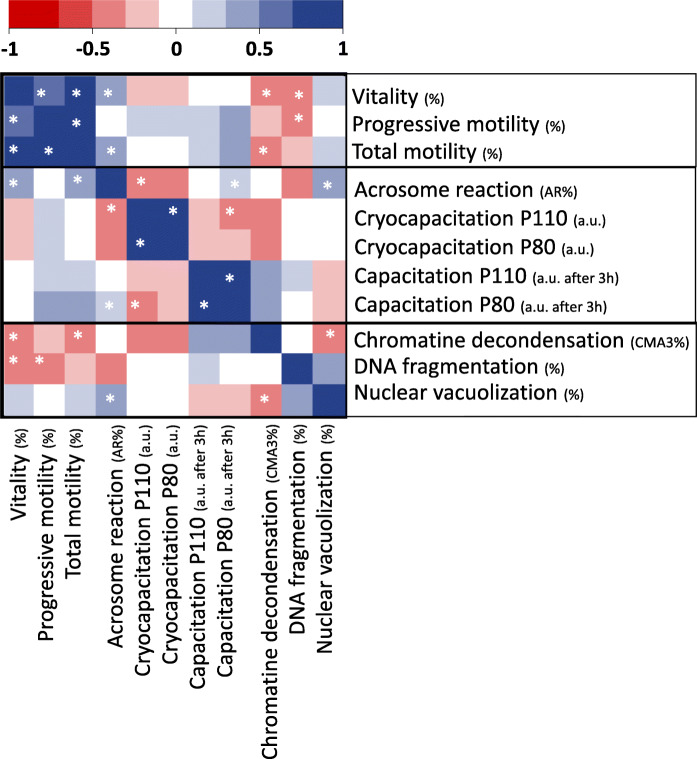
Correlations between post thaw spermatozoa parameters. Heat map showing the positive (blue) and negative (red) correlations between the different parameters using Spearman correlations. *indicates a significant correlation with *p* < 0.05

With regard to sperm nuclear quality, chromatin packaging was significantly higher in the H+ arm than in the H- arm, from use of CMA3 labelling (− 16.0% spermatozoa with decondensed chromatin, ES = − 0.30 [− 0.57; − 0.03], *p* < 0.05, Fig. 3A). Sperm chromatin decondensation was significantly was reduced by hypotaurine supplementation in abnormal samples (− 20.8% *p* < 0.05) but not in normal ones (10.7 ± 1.0 for H- vs. 9.6 ± 1.5% for H+). Similar effects were observed with the percentages of spermatozoa with DNA fragmentation (− 18.7 ± 2.0% relative to the “H-” arm, ES = − 0.37 [− 0.80; − 0.02] *p* < 0.05, Fig. 3B) and nuclear vacuolization (− 20.8%, ES = − 0.34 [− 0.68; − 0.03], *p* < 0.05, Fig. 3C). The nuclear alterations of the spermatozoa were correlated with each other and with the different parameters measured (heatmap, Fig. 4). The percentage of CMA3+ spermatozoa was negatively correlated with the percentage of spermatozoa with nuclear vacuoles (r = − 0.4, *p* < 0.01), vitality (r = − 0.4, *p* < 0.01) and total motility (r = − 0.4, *p* < 0.01). The percentage of spermatozoa with fragmented DNA was negatively correlated with vitality (r = − 0.5, *p* < 0.01) and progressive motility (r = − 0.4, *p* < 0.05). The percentage of spermatozoa with nuclear vacuolization was positively correlated with the percentage of acrosome reacted spermatozoa (r = 0.4, *p* < 0.05).

## Discussion

Despite the development of new tools and approaches for sperm cryopreservation, slow freezing remains the gold standard method for preserving male fertility [2, 3, 5]. However, sperm preparations including washing, DGC selection, as well as cryopreservation itself, are known to increase oxidative stress, leading to the alteration of the sperm membrane, its nucleus and the DNA it contains [9, 35]. In addition, DGC, removing the protective AO activities of the seminal fluid may increase the susceptibility of spermatozoa to oxidative damage [19]. In this context, the addition of AO during DGC and cryopreservation processes could have a beneficial effect on sperm structures and functions. Our previous study [28] reported a decrease in the percentage of spermatozoa with apoptotic markers when sperm selection was performed before freezing with the addition of hypotaurine. The present study clearly demonstrated the benefits of the combination of DGC and hypotaurine supplementation on spermatozoa vitality, total and progressive motility, cryo-capacitation and DNA integrity after thawing in patients with normal or abnormal conventional sperm parameters. In particular, this protocol led to a significant reduction of the proportion of spermatozoa with nuclear alterations such as chromatin decondensation, DNA fragmentation, and nuclear vacuolization.

In addition to its well-known osmoregulatory action, hypotaurine has been described as an antioxidant [36, 37]. Both hypotaurine and taurine, its immediate downstream metabolite, are naturally present in male and female human reproductive tissue. Both substances have long been reported to be related to male fertility [37–39]. They have been shown to play a role in various processes, including protective effects on motility [37, 40–42]. As the freezing medium already contains taurine in its original composition, only hypotaurine was added in the different media. Hypotaurine supplementation in the media has already shown beneficial effects on sperm function using animal models including ram, dog, golden hamster, rabbit, chicken and bull. It has improved the integrity of sperm plasma and acrosomal membranes [41, 43], reducing sperm lipid peroxidation [38, 42]. In addition, hypotaurine supplementation has been reported to decrease OS [36] and DNA fragmentation [41, 42] particularly in the context of cryopreservation [41, 42, 44, 45]. Finally, hypotaurine has been reported to improve to ability of spermatozoa to undergo capacitation, the acrosome reaction and fertilization [37, 45–47]. Therefore, we hypothesized that because of its combined antioxidant, anti-capacitant and osmoregulatory actions, hypotaurine supplementation of the DGC, washing and freezing media may be an appropriate choice for improving the quality of cryopreserved human spermatozoa [2, 7, 28]. A recent study has published results consistent with ours, showing the beneficial effects of hypotaurine supplementation during the vitrification of human spermatozoa, including improved acrosomal integrity, motility, morphology and reduced lipid peroxidation [48].

All measured nuclear alterations were significantly reduced by hypotaurine supplementation. The protective effects of hypotaurine on sperm nuclear integrity reported here confirmed our preliminary report [28] and are consistent with other reports that have shown that AO supplementation in freezing media protects sperm cells from DNA damage [2, 5, 23]. Our study demonstrates that the addition of AO to the selection and freezing media reduces both sperm nuclear vacuolization and alterations in chromatin packaging. In addition, we showed in the present study that sperm nuclear vacuolization was positively correlated with alterations in acrosomal integrity, but negatively with chromatin decondensation.

Hypotaurine reduced the non-specific phosphorylation of two capacitation markers induced by cryopreservation in the process known as cryo-capacitation, but had no significant impact on the integrity of the acrosome. Hypotaurine supplementation has been reported [41] in the ram model to reduce the deleterious effects of cryopreservation on the acrosome. However, Partyka et al. showed no effect on the acrosome integrity of chicken spermatozoa [42]. The action of hypotaurine appears to be dose-dependent and possibly species-specific. The 50 mM concentration was chosen on the basis of concentrations measured in the seminal and epididymal fluids of fertile men [37]. This concentration was also the highest used in this context compared to other studies [41, 42]. Our objective was to stay in a physiological concentration in order to avoid deleterious effect of antioxidant excess. It has been shown that unsuitable or excessive antioxidant supplementation can have harmful effects, in particular on chromatin condensation [49]. Our strategy could explain, at least in part, that the beneficial effect of hypotaurine was more marked for abnormal sperm for vitality and motility. Studies showed that semen from infertile men with oligoasthenoteratozoospermia contained lower hypotaurine concentration [37, 50] and that spermatozoa from these patients could be less protected against cryopreservation-induced OS [28]. Dose-response experiments will be conducted to determine which concentrations could possibly further enhance the effects of hypotaurine on human spermatozoa. In addition, hypotaurine could not be efficient to counteract all oxidative damages notably those mediated by H_2_O_2_. Indeed, publications showed that hypotaurine can scavenge the aggressive hydroxyl radical [36, 51], but its ability to deal with H_2_O_2_ (or/and H_2_O_2_-mediated damage) is not clearly established. It has recently been shown that different antioxidants, when combined, can have additive positive effects on spermatozoa (for recent reviews [2, 5, 22, 23]). Other AO will be tested in combination with hypotaurine to measure potential synergistic and/or complementary action. Numerous AO had been already tested in combination with hypotaurine such as glutathione [51], taurine [44, 45, 48] and L-carnitine [42]. The most current and efficient combination was with taurine, that was already present in the cryopreservative medium used. It is probable that the effects observed were the consequence of the combination of taurine and hypotaurine. Others AO could be envisaged [23, 52] such as selenium, zinc, vitamins C and E and also curcumin [53] for which beneficial effects have recently been shown during human sperm cryopreservation.

## Conclusion

We have shown here that human sperm samples selected by DGC and frozen in the presence of hypotaurine had, post-thawing, a significantly higher proportion of live and progressively motile spermatozoa, compared to semen that were selected solely by DGC before freezing. Hypotaurine supplementation limited cryo-capacitation and reduced the percentage of spermatozoa showing chromatin decondensation, DNA fragmentation and nuclear vacuolation. Sperm cryopreservation is a valuable clinical tool for the management of male preservation fertility. Recently, the Cochrane review [52] concluded that there is poor evidence of the beneficial effects of oral antioxidant supplementation for subfertile men. We show here that hypotaurine supplementation could be easily and routinely added to improve the sperm freezing protocols.

## Data Availability

The datasets during and/or analysed during the current study available from the corresponding author on reasonable request.

## Abbreviations

AO: antioxidant
AR: acrosome reacted
BMI: body mass index
BSA: bovin serum albumin
C.I.: confidence interval
CMA3: chromomycin A3
CPP: Committee for Personal Protection
°C: degree Celsius
DGC: density gradient centrifugation
DNA: deoxyribonucleic acid
FCM: flow cytometry
Fig: Figure
FITC: fluorescein isothiocyanate
FSC: forward-scattered light
h: hour
IRB: Institutional Review Board
OAT: oligo-astheno-teratozoospermia
MSOME: motile sperm organelle morphology examination
min: minute
OS: oxidative stress
%: percent
PBS: phosphate buffered saline
PMN: PolyMorphonuclear Neutrophil
PI: Propidium Iodide
PSA: *P. sativum* agglutinin
P-Tyr: tyrosine phosphorylation
ROS: reactive oxygen species
RT: room temperature
SSC: side-scattered light
SEM: Standard Error of the Mean
Spz: spermatozoa
WHO: World Health Organization
